# A survivin-responsive, conditionally replicating adenovirus induces potent cytocidal effects in adult T-cell leukemia/lymphoma

**DOI:** 10.1186/s12885-019-5730-1

**Published:** 2019-05-29

**Authors:** Shinsuke Suzuki, Hiroki Kofune, Kimiharu Uozumi, Makoto Yoshimitsu, Naomichi Arima, Kenji Ishitsuka, Shin-ichi Ueno, Ken-ichiro Kosai

**Affiliations:** 1 Department of Clinical Oncology, Course of Advanced Therapeutics, Kagoshima University Graduate School of Medical and Dental Sciences, 8-35-1 Sakuragaoka, Kagoshima, 890-8520 Japan; 2 Department of Hematology and Rheumatology, Kagoshima University Hospital, Kagoshima, Japan; 3 Department of Medical Oncology, National Hospital Organization Kagoshima Medical Center, Kagoshima, Japan; 4 Department of Gene Therapy and Regenerative Medicine, Course of Advanced Therapeutics, Kagoshima University Graduate School of Medical and Dental Sciences, Kagoshima, Japan

**Keywords:** ATL, Survivin, Conditionally replicating adenoviruses

## Abstract

**Background:**

Adult T-cell leukemia/lymphoma (ATL) is a peripheral T-cell malignancy caused by long-term human T-cell leukemia virus type I (HTLV-1) infection. Survivin-responsive, conditionally replicating adenoviruses regulated by multiple tumor-specific factors (Surv.m-CRAs), in which the expression of the adenoviral early region 1A gene is regulated by the survivin (*BIRC5*) promoter, can be used to treat several cancers. As survivin is overexpressed in ATL, we examined the effects of Surv.m-CRAs on ATL-selective replication and survival.

**Methods:**

We tested two ATL cell lines and four HTLV-1-infected T-cell lines. The cells were subjected to infection with either E1-deleted, replication-defective adenoviruses or Surv.m-CRAs at various multiplicities of infection.

**Results:**

Strong activation of survivin promoter was observed in all six cell lines. Moreover, the expression of the coxsackie and adenovirus receptor (CAR), which is important for adenoviral infection, was high in the cell lines. In contrast, we observed the absence of survivin promoter activity and a low expression of CAR in activated peripheral blood lymphocytes (PBLs) from healthy subjects. Surv.m-CRAs actively replicated and induced cytocidal effects in five out of six cell lines; conversely, we observed minimal viral replication and no marked cytotoxicity in normal activated PBLs.

**Conclusions:**

This is the first report demonstrating that Surv.m-CRAs constitute attractive potential anti-ATL agents.

## Background

Adult T-cell leukemia/lymphoma (ATL) is an aggressive malignancy of mature peripheral T lymphocytes that results from long-term infection with human T-cell leukemia virus type I (HTLV-1). Five to twenty million individuals are estimated to be currently infected with the virus, and only 3–5% of HTLV-1-infected patients develop ATL. HTLV-1 infection can lead to the insertion of oncogenic viral genes into the host genome, which activate NF-κB and Akt signaling, as well as cyclin-dependent kinases, and silence TP53 in host cells.

The prognosis for patients with ATL after treatment with classical cytotoxic agents is still poor. New therapeutic approaches, distinct from standard cytotoxic chemotherapy treatments, are expected to lead to the eradication of ATL [1, 2]. For example, the efficacy of combined interferon-alpha and zidovudine treatment for aggressive ATL has been described in some uncontrolled clinical studies [3, 4]. A meta-analysis revealed that compared to chemotherapy alone, first-line management with interferon-alpha and zidovudine was markedly more effective for patients with acute-type ATL [5]. As an immunotherapeutic approach that can induce long-term survival, allogeneic hematopoietic stem cell transplantation (HSCT) is recurrently conducted for the treatment of aggressive ATL in Japan. The main problem with allogeneic HSCT is that mortality rate related to treatment is high, affecting up to 40% of patients [6–8]. Some promising therapeutic advances in the management of patients with ATL in Japan include the introduction of the anti-CCR4 monoclonal antibody, mogamulizumab, and the immunomodulatory drug, lenalidomide [9, 10].

Conditionally replicating adenoviruses (CRAs), also termed oncolytic adenoviruses, which selectively replicate in and eradicate cancer cells, are smart anti-cancer agents [11]. We developed a unique vector, which is regulated by multiple tumor-specific factors (m-CRA) [11, 12], by additional modification of a CRA derived from a human adenovirus. Both cancer specificity and effective viral replication are crucial for any CRA-based therapy. Early region 1A (*E1A*), the first transcribed gene after infection with wild-type adenoviruses, transactivates the viral and cellular genes essential for generating transmittable adenoviruses. m-CRAs replicate in a tumor-specific manner by replacing the innate *E1A* promoter with a tissue- and tumor-specific promoter [13–15].

Survivin (BIRC5), a member of the inhibitor of apoptosis gene family, is overexpressed in most cancers, but not in normal cells. Clinical trials have demonstrated a positive correlation between high survivin expression levels and poor prognosis, enhanced rate of recurrence, and increased resistance to chemotherapy in patients with malignancy [16].

We developed survivin-responsive m-CRAs (Surv.m-CRAs), which express E1A under the transcriptional control of the survivin promoter. Surv.m-CRAs can kill a variety of cancers more efficiently and specifically than telomerase-responsive m-CRAs in vitro and in vivo [17]. Moreover, Surv.m-CRAs can kill cancer stem cells and tumorigenic pluripotent stem cell populations, which are often resistant to classical cytotoxic agents [18, 19].

As survivin is overexpressed in ATL, especially in acute-type ATL [20], we examined the effects of Surv.m-CRAs on ATL-selective replication and the induction of cytocidal activity, with the ultimate goal of applying Surv.m-CRA clinically for the treatment of ATL in the future.

## Methods

### Cell lines

The human ATL cell lines, S1T and Su9T01, and the HTLV-1-infected T-cell lines, Oh13T, K3T, F6T, and MT-2, were sustained in RPMI 1640 (Gibco/Invitrogen, Carlsbad, CA, USA) supplemented with 1% penicillin/streptomycin (Gibco/Invitrogen) and 10% fetal bovine serum (Gibco/Invitrogen). All the cell lines, except MT-2, were established from patients in our laboratory [21]. MT-2 was purchased from Japanese Cancer Research Resources Bank (Osaka, Japan; catalogue number: JCRB1210). We used activated peripheral blood lymphocytes (PBLs) from healthy subjects as negative controls. The PBLs were incubated in the presence of 10 U/ml recombinant human IL-2 (Amgen Biologicals, Thousand Oaks, CA, USA) for 6 days at 37 °C in a 95%-humidity atmosphere with 5% CO_2_. Written informed consent was obtained from healthy subjects to participate of this research.

### Adenoviruses

The following viruses were proliferated and purified as described previously [17]: Surv.m-CRA, with the wild-type *E1A* under the transcriptional control of the survivin promoter, E1B55KD under the transcriptional control of the CMV promoter, and *EGFP* under the transcriptional control of the CMV promoter; two types of E1-deleted, replication-defective adenoviruses expressing *EGFP* under the transcriptional control of the CMV promoter (Ad.CMV-EGFP) or *LacZ* under the transcriptional control of the survivin promoter (Ad.Surv-LacZ) were also proliferated and purified.

### Promoter activities and AGTE

Promoter activities and in vitro adenoviral gene transduction efficiency (AGTE) were examined as described previously with some modifications [17]. Cells (5 × 10^5^/plate) were infected with Ad.Surv-LacZ at a multiplicity of infection (MOI) of 30 for 24 h. We measured cellular β-gal activity using the β-Galactosidase Enzyme Assay System (Promega, Madison, WI, USA), after harvesting. The AGTE for each cell type in vitro was examined 48 h after infection with Ad.CMV-EGFP at an MOI of 30.

### Flow cytometric analysis

We analyzed the percentage of EGFP- and PE-positive cells by flow cytometry on a FACSCalibur™ (Becton Dickinson, San Jose, CA, USA). Using flow cytometry, we assessed the expression of coxsackie and adenovirus receptor (CAR), which is recognized as the primary cropping receptor for coxsackie B viruses and members of the adenovirus family JM Bergelson, JA Cunningham, G Droguett, EA Kurt-Jones, A Krithivas, JS Hong, MS Horwitz, RL Crowell and RW Finberg [22]. Cells were stained with RmcB (Sigma Immunochemicals, St. Louis, MO, USA), the primary antibody against CAR, or an isotype control for 60 min on ice. After washing and centrifugation, cells were incubated for 30 min on ice with PE-conjugated goat anti-mouse immunoglobulin (F (ab′)2 fragment; DAKO A/S, Glostrup, Denmark). Cells were resuspended at 1.0 × 10^7^/ml in phosphate buffered saline, and then kept on ice until analysis.

### MTT reduction assay

Cells in 96-well plates were infected with Surv.m-CRA at MOIs of 0.3, 3, and 30 or Ad.CMV-EGFP at an MOI of 30. Cell viability was determined by MTT assay (Sigma–Aldrich, St. Louis, MO, USA) after 3 and 5 days, according to the manufacturer’s protocol.

### Statistical analysis

Data are shown as the mean ± standard errors (s.e.). Statistical significance was determined using Student’s *t*-test. *P* < 0.05 was concluded statistically significant.

## Results

**Fig. 1:**
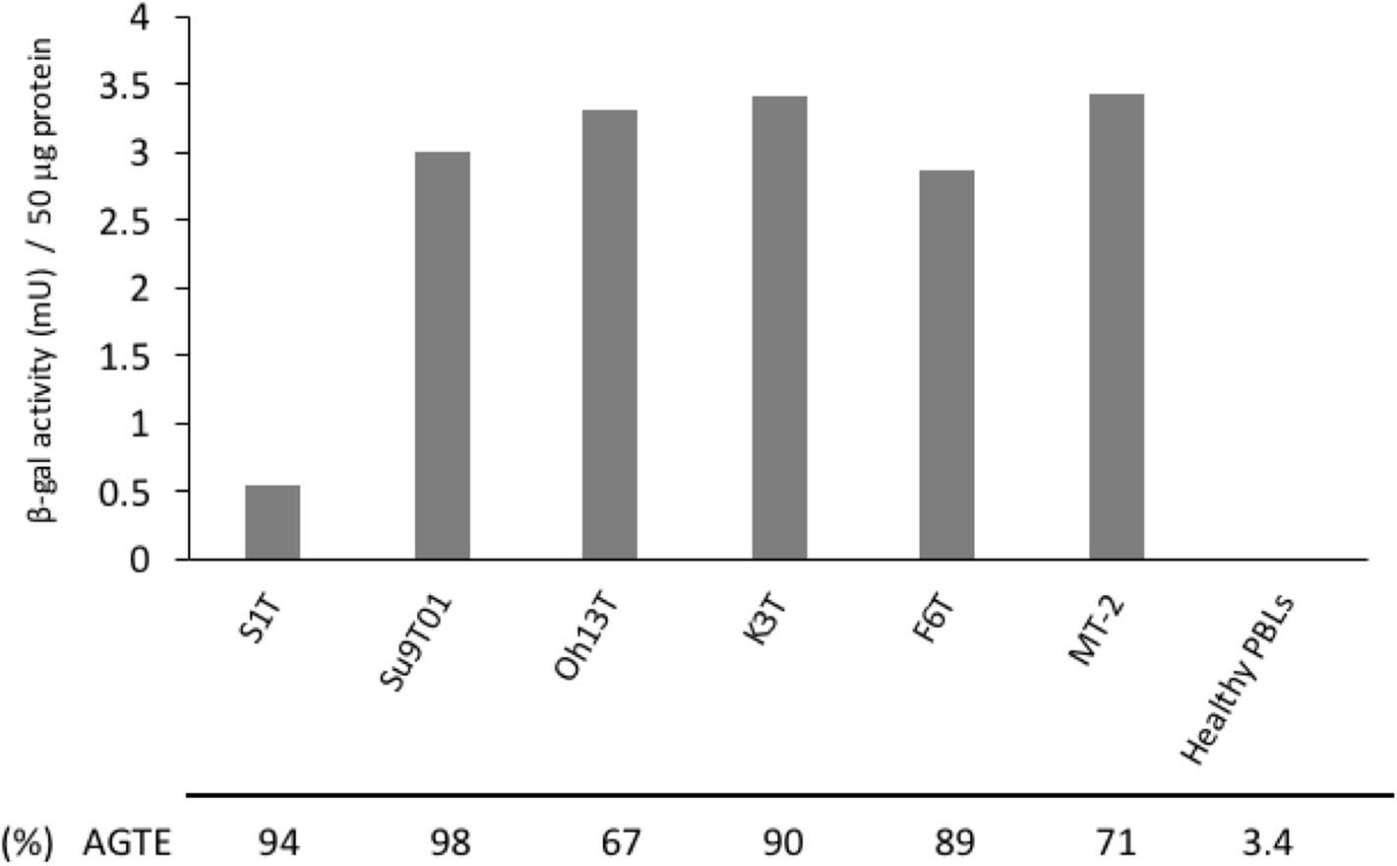
Assessment of survivin promoter activity and AGTE after infection. β-gal enzyme activity was detected 48 h after infection with Ad.Surv-LacZ at an MOI of 30. Columns indicate mean of three independent experiments. AGTE is presented as the percentage of EGFP-stained cells observed among the total cells at 48 h after Ad.CMV-EGFP infection at an MOI of 30. The activated peripheral blood lymphocytes (PBLs) from healthy subjects were used as negative controls

We tested 2 ATL cell lines and 4 HTLV-1-infected T-cell lines. We infected the cells with Ad.CMV-EGFP or Ad.Surv-LacZ at an MOI of 30. All the cell lines had high AGTE and strong survivin promoter activation (Fig. 1). As opposed to its high AGTE (94%), S1T had lower β-gal activity (i.e. survivin promoter activity) after infection than the other cell lines. Importantly, we did not detect β-gal activity in healthy activated PBLs after infection with the adenoviruses (Fig. 1). This was likely due to their low AGTE (3.4%) and low survivin promoter activity. Thus, survivin promoter activity was markedly increased in the cell lines, despite small variations in activation, in comparison to the activity in normal PBLs.

**Fig. 2:**
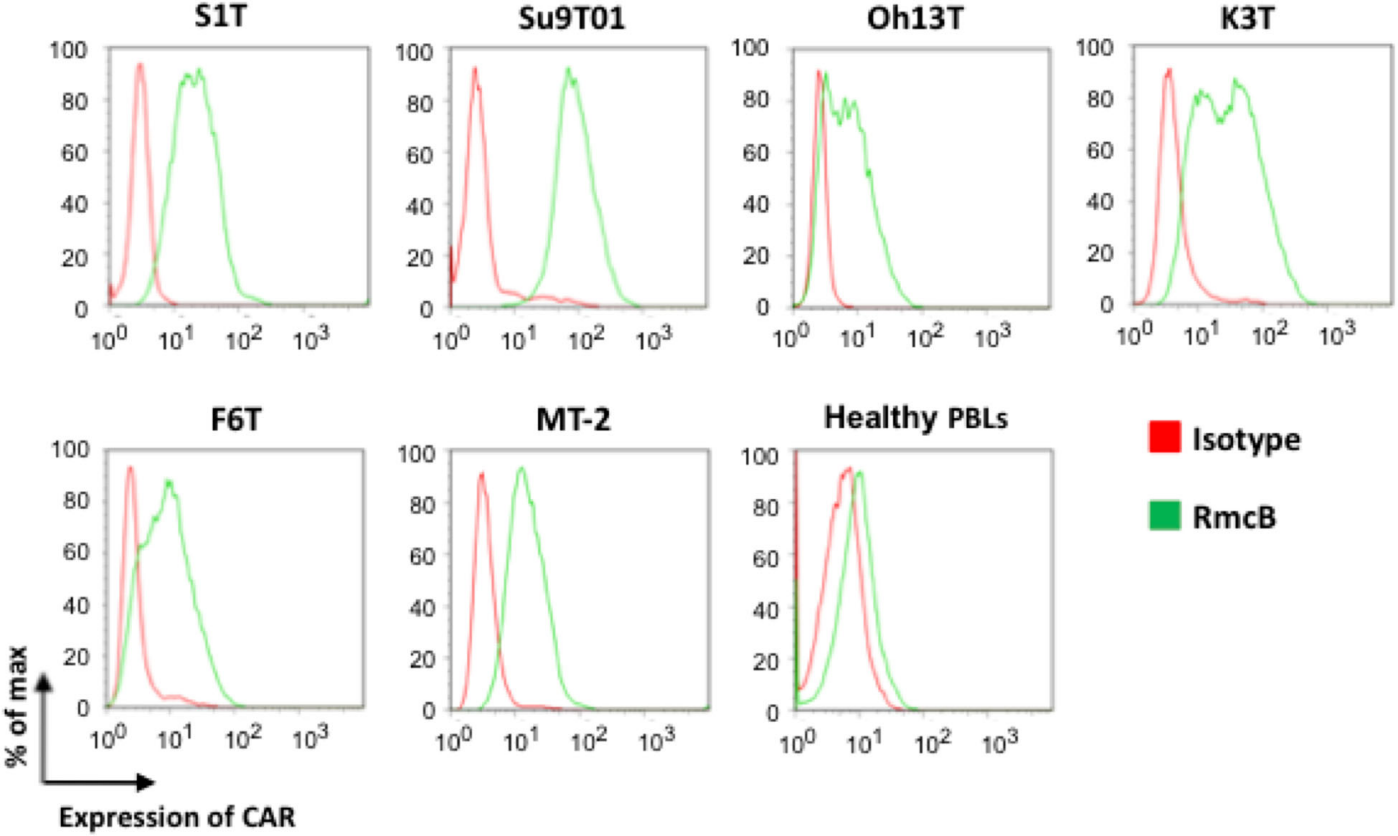
Flow cytometric analysis of CAR expression in ATL cell lines and HTLV-1-infected T-cell lines. The expression of CAR was analyzed in ATL cell lines, S1T and Su9T01, and the HTLV-1-infected T-cell lines, Oh13T, K3T, F6T, and MT-2. CAR was labelled with the primary antibody RmcB or with an isotype control followed by a PE-linked secondary antibody

**Fig. 3:**
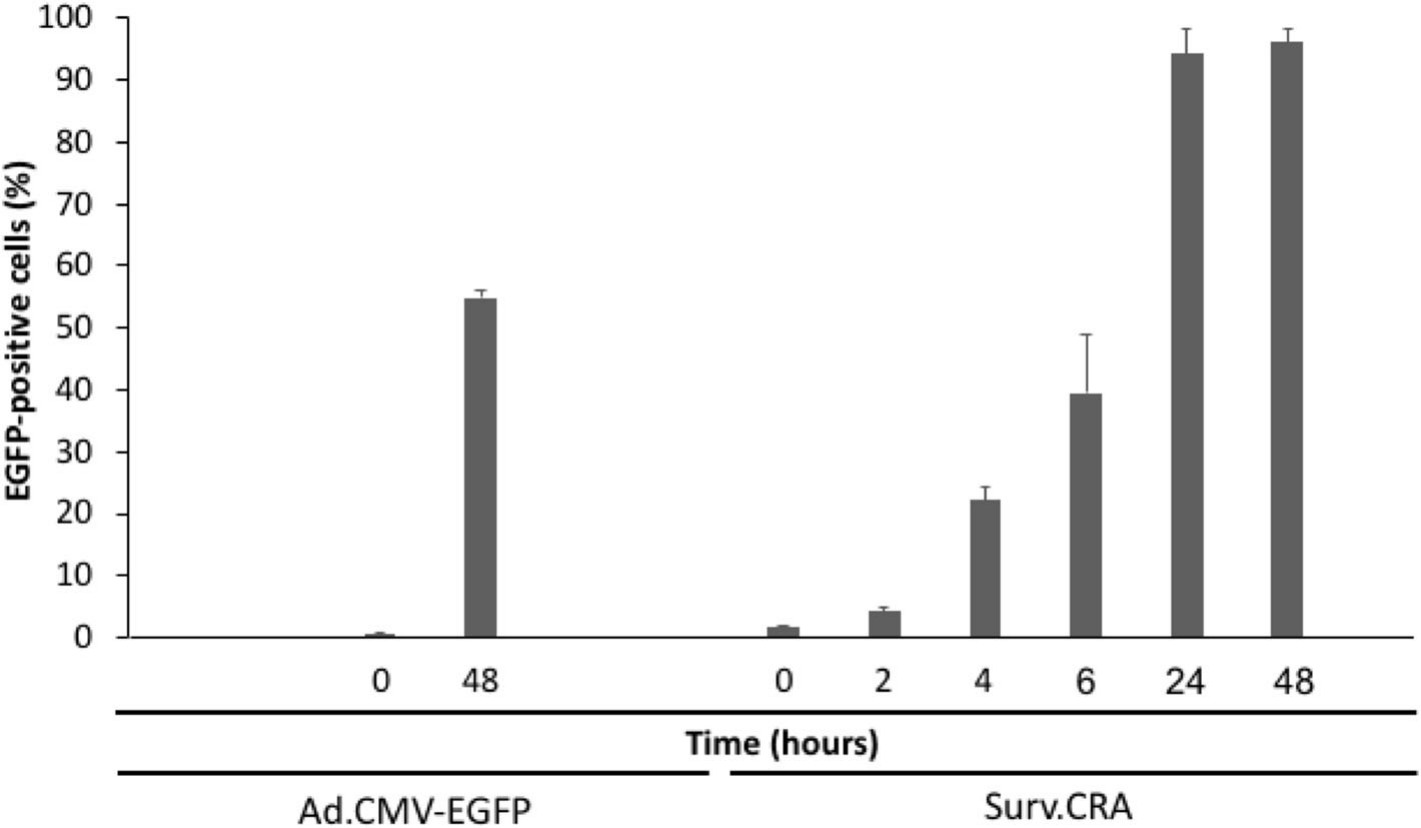
Replication of Surv.m-CRA in the cell line K3T. EGFP-positive cells increased in a time-dependent manner after infection with Surv.m-CRA. The percentage of EGFP-stained cells observed among the total cells at 48 h after Ad.CMV-EGFP or Surv.m-CRA infection at an MOI of 3 is 55.3 and 96.1%, respectively. Each bar represents the mean of 3 independent experiments

Moreover, CAR, a strong, but not perfect, determinant for cell infection by adenoviruses, was highly expressed in the cell lines. In contrast, low expression of CAR was observed in activated PBLs from healthy subjects (Fig. 2). The survivin promoter induced strong transcriptional activation in the K3T cell line, which showed sufficient viral replication. The frequency of EGFP-positive cells increased in a time-dependent manner after infection with Surv.m-CRA. The percentage of EGFP-stained cells observed among the total K3T cells at 48 h after Surv.m-CRA or Ad.CMV-EGFP infection at an MOI of 3 was 96.1 and 55.3%, respectively. These results indicated that Surv.m-CRA replicated in nearly all of the cells (Fig. 3).

**Fig. 4:**
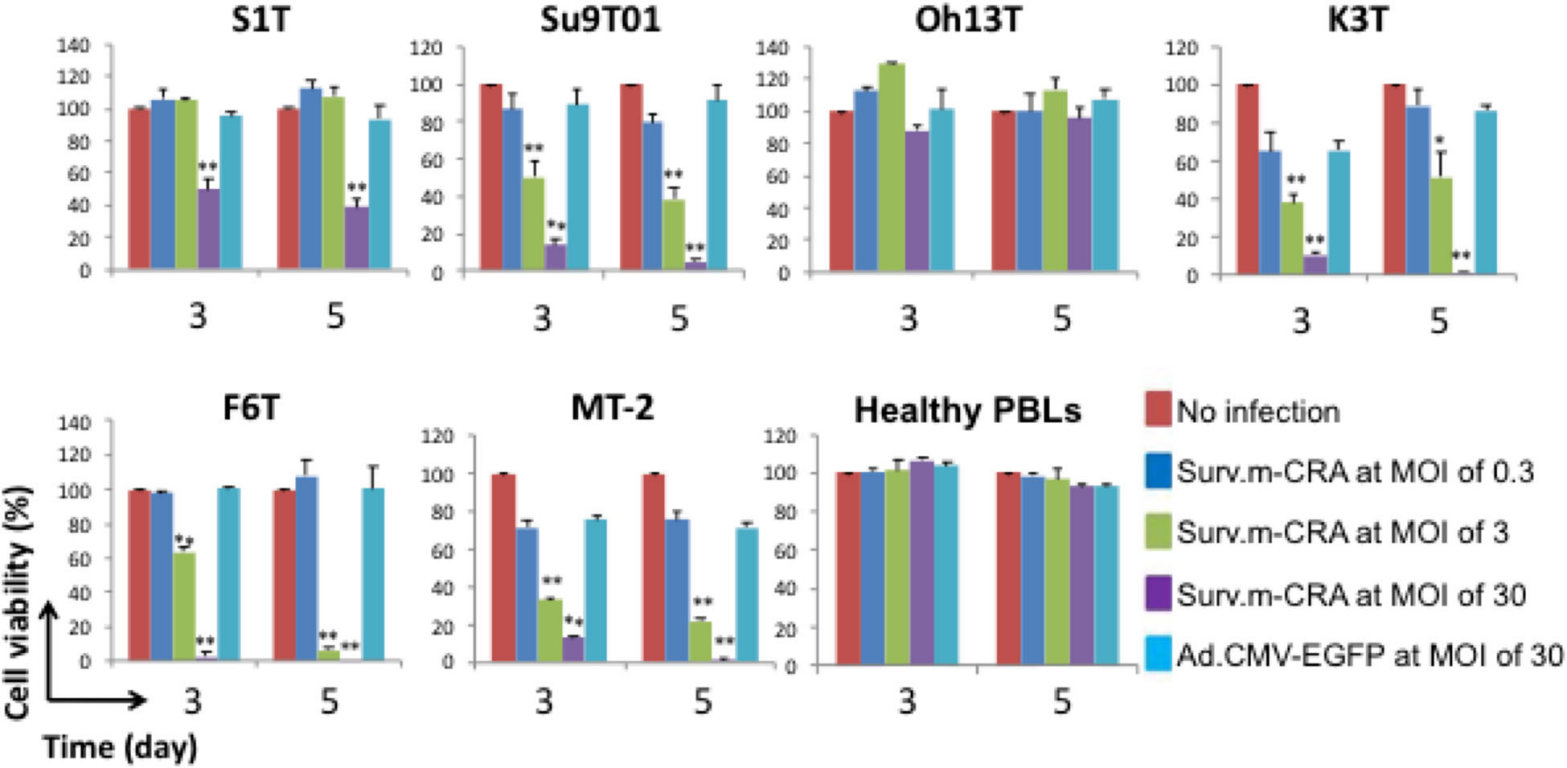
Cytotoxic effects of Surv.m-CRA in ATL cell lines and HTLV-1-infected T-cell lines. Cells were infected with Surv.m-CRA at MOIs of 0.3, 3, and 30 or Ad.CMV-EGFP at an MOI of 30. Cell viability was determined by MTT assay at day 3 or 5 after infection. Each bar represents the mean of three independent experiments. *P < 0.05 and **P < 0.001 (statistical significance in comparison with the Ad.CMV-EGFP group)

Surv.m-CRAs efficiently replicated and effectively induced cell death in 5 of 6 cell lines; there was a slight viral replication in normal activated PBLs and no significant cytotoxicity (Fig. 4). Thus, both a strong cytotoxic effect and strict tumor specificity were observed in this setting.

## Discussion

One of the most appealing features of Surv.m-CRAs is their capacity to target a variety of malignant tumors [1]. This is the first report indicating that Surv.m-CRAs constitute a potential anti-leukemia/lymphoma agent. We demonstrated that Surv.m-CRAs efficiently replicated and induced potent cytocidal effects in ATL and HTLV-1-infected T-cell lines, but not in normal activated PBLs. This finding was consistent with previous reports that survivin mRNA is overexpressed in ATL [20].

In this study, all the ATL and HTLV-1-infected T-cell lines had high AGTEs and survivin promoter activity. Surv.m-CRAs potently induced cytotoxicity in 5 of the 6 cell lines.

Low AGTE in certain cancer types is a serious issue in adenoviral gene therapy; Surv.m-CRAs are no exception [17]. Surprisingly, all the ATL and HTLV-1-infected T-cell lines had high AGTEs and high CAR expression, with low AGTEs and low CAR expression in activated PBLs. Other investigators reported that CAR expression was high in some lymphoid malignancies, e.g. in cells derived from the bone marrow of patients with multiple myeloma and in a cell line derived from anaplastic large cell lymphoma. They hypothesized that the malignant transformation process of myeloma and lymphoma closely correlates with the expression of CAR [23, 24]. Whether this characteristic occurs in common lymphoid tumors or it is peculiar to ATL, multiple myeloma, and some anaplastic large cell lymphoma is not yet known. Further investigation is warranted to elucidate the underlying molecular mechanism responsible.

High CAR expression is a strong, but not perfect, determinant for cell infection by adenoviruses. Actually, some solid tumor cell lines cause adenovirus infection at a relatively high rate even with CAR deficiency; however, the mechanism has not yet been fully elucidated. However, our results suggested that adenoviral gene therapy holds potential for the treatment of ATL caused by HTLV-1 infection.

Another critical requirement for ideal CRA is attenuation of viral replication in normal cells. We have already demonstrated that survivin promoter activity is not present in normal WI-38 fibroblasts, despite high levels of CMV promoter activity and moderate to high levels of AGTE. Surv.m-CRAs could not induce cytotoxic effects in either normal activated PBLs with low AGTE levels or WI-38 cell lines, indicating that moderate to high AGTE was not sufficient for cytotoxicity of Surv.m-CRAs [17]. Moreover, we previously showed that a single injection of Surv.m-CRAs into a pre-established tumor, expressing survivin, induced significant tumor death and inhibited tumor growth. These results suggest the therapeutic potential and safety of Surv.m-CRAs for the treatment of cancer [17]. Fukuda et al. showed that survivin is expressed and regulated by growth factors in human CD34+ cells, which are considered hematopoietic stem cells (HSCs) [25]. Although the paucity of CAR is thought to be a limiting factor for Surv.m-CRA transfer into HSCs, myelosuppressive side effects must be considered in clinical settings [26].

**Fig. 5:**
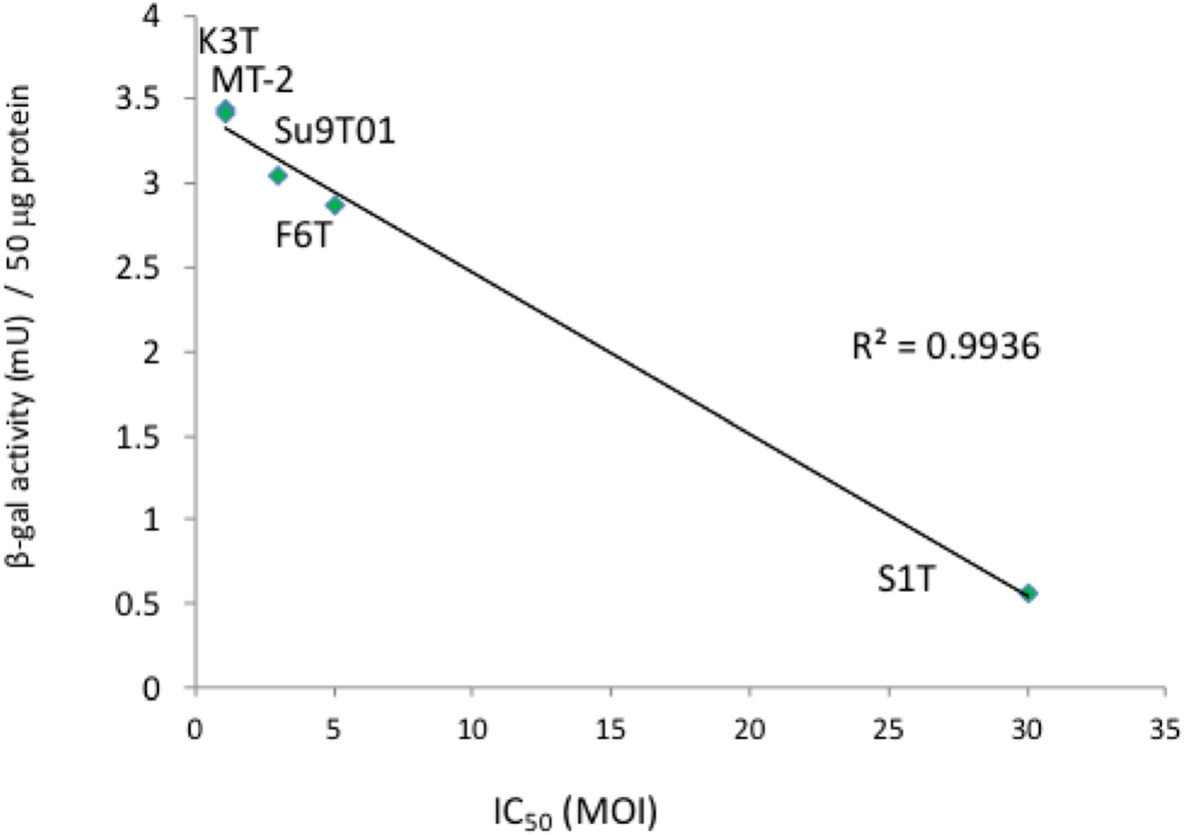
Relationship between survivin promoter activity and the cytotoxic effects of Surv.m-CRA. We examined the relationship between the survivin promoter activity of CRAs in ATL cell lines and HTLV-1-infected T-cell lines and the IC_50_ of Surv.m-CRAs in the cell lines. The coefficient of determination, denoted as R^2^, is 0.99356. Oh13T, which exhibited complete resistance to Surv.m-CRA treatment, was excluded from this analysis

Among the ATL and HTLV-1-infected T-cell lines, only Oh13T cells exhibited complete resistance to Surv.m-CRA treatment. This exceptional result must be further explored to understand the mechanism of resistance. The growth rate of the Oh13T cells was slower than that of the other cell lines, which may confer resistance to the cytocidal effects of Surv.m-CRAs. Although multiple factors are involved in Surv.m-CRA therapy, high survivin promoter activity is a promising, but not perfect, biomarker that correlated with the efficacy of Surv.m-CRA therapy in the other tested cell lines (Fig. 5).

Although our experiments do not include any in vivo therapeutic data, this is the first report demonstrating that Surv.m-CRAs show an apparent difference in AGTE, CAR expression, promoter activity, and cytocidal effect between hematologic malignancy and activated PBLs. Moreover, we are currently implementing a first-in-human, investigator-initiated trial of Surv.m-CRAs for the treatment of refractory malignant bone and soft tissue tumors. Our results suggest the possibility of using Surv.m-CRAs for the clinical treatment of ATL based on the clinical trial with the conventional solid tumors. However, unlike solid tumors, there are several possible gene transduction methods for the delivery of adenoviral gene against leukemic cells. In future extensive studies, we have to focus on the methods of gene administration.

Another attractive feature of Surv.m-CRAs is their ability to target cancer stem cells. Surv.m-CRAs demonstrate elevated effectiveness against cancer stem cells, which are often resistant to conventional anti-cancer medications and radiotherapy [18]. We are currently implementing a first-in-human, investigator-initiated trial of Surv.m-CRAs for the treatment of refractory malignant bone and soft tissue tumors.

Recently, immunotherapies using antibodies against programmed cell death 1 (PD-1) and its ligand (PD-L1) have proven to be powerful and effective treatments for patients with various advanced cancers in many clinical trials [27–30]. The most attractive feature of oncolytic virus therapy is that systemic, tumor-specific immunity is efficiently induced via the oncolytic activity of the viruses [31]. As viruses are also known to activate high-affinity, antigen-specific cytotoxic T lymphocytes against non-self viral antigens, cytolytic activity is enhanced against host cancer cells that express transcripts from Surv.m-CRAs. Talimogene laherparepvec was approved for the treatment of melanoma by the United States Food and Drug Administration in October 2015. A phase III trial first proved that local, intralesional injections of an oncolytic virus can not only suppress the growth of the injected tumors but also prolong overall survival, reportedly via the induction of systemic anti-tumor immunity [32]. However, Rooney et al. suggested that viruses are likely to drive cytolytic activity, and they revealed known and novel mutations that enable viral-associated tumors to resist immune attack [33]. In fact, Kataoka et al. revealed a unique genetic mechanism of immune escape caused by structural variations that commonly disrupt the 3′ region of the PD-L1 (*CD274*) gene in multiple cancers, especially in ATL. PD-L1 disruption could serve as a genetic marker to identify cancers that actively evade anti-tumor immunity through PD-L1 overexpression [34]. Combination treatment with systemic administration of PD-1/PD-L1 blockade is a reasonable strategy to enhance the efficacy of oncolytic viruses against ATL.

## Conclusions

This is the first report demonstrating that Surv.m-CRAs represent a striking potential anti-leukemic agent that could efficaciously and specifically treat ATL. Because our experiments did not include any in vivo therapeutic data, we will focus on the methods of gene administration in future extensive studies.

## Data Availability

All data generated or analyzed during this study are included in this published article.

## Abbreviations

Ad.CMV-EGFP: E1-deleted, replication-defective adenoviruses, expressing EGFP under the transcriptional control of the CMV promoter;
Ad.Surv-LacZ: E1-deleted, replication-defective adenoviruses, expressing LacZ under the transcriptional control of the survivin promoter
AGTE: Adenoviral gene transduction efficiency
ATL: Adult T-cell leukemia/lymphoma
CAR: Coxsackie and adenovirus receptor
CRAs: Conditionally replicating adenoviruses
E1A: Early region 1A
HSCs: Hematopoietic stem cells
HSCT: Hematopoietic stem cell transplantation
HTLV-1: Human T-cell leukemia virus type I
m-CRA: Multiple tumor-specific factors
MOI: Multiplicity of infection
PBLs: Peripheral blood lymphocytes
PD-1: Programmed cell death 1
PD-L1: Programmed cell death ligand 1
Surv.m-CRAs: Survivin-responsive, conditionally replicating adenoviruses regulated by multiple tumor-specific factors

## References

[1] Ishitsuka K, Tamura K (2014). Human T-cell leukaemia virus type I and adult T-cell leukaemia-lymphoma. Lancet Oncol.

[2] Uozumi K (2010). Treatment of adult T-cell leukemia. J Clin Exp Hematop.

[3] Gill PS, Harrington W, Kaplan MH, Ribeiro RC, Bennett JM, Liebman HA, Bernstein-Singer M, Espina BM, Cabral L, Allen S (1995). Treatment of adult T-cell leukemia-lymphoma with a combination of interferon alfa and zidovudine. N Engl J Med.

[4] Hermine O, Bouscary D, Gessain A, Turlure P, Leblond V, Franck N, Buzyn-Veil A, Rio B, Macintyre E, Dreyfus F (1995). Brief report: treatment of adult T-cell leukemia-lymphoma with zidovudine and interferon alfa. N Engl J Med.

[5] Bazarbachi A, Plumelle Y, Carlos Ramos J, Tortevoye P, Otrock Z, Taylor G, Gessain A, Harrington W, Panelatti G, Hermine O (2010). Meta-analysis on the use of zidovudine and interferon-alfa in adult T-cell leukemia/lymphoma showing improved survival in the leukemic subtypes. J Clin Oncol.

[6] Fukushima T, Miyazaki Y, Honda S, Kawano F, Moriuchi Y, Masuda M, Tanosaki R, Utsunomiya A, Uike N, Yoshida S (2005). Allogeneic hematopoietic stem cell transplantation provides sustained long-term survival for patients with adult T-cell leukemia/lymphoma. Leukemia.

[7] Hishizawa M, Kanda J, Utsunomiya A, Taniguchi S, Eto T, Moriuchi Y, Tanosaki R, Kawano F, Miyazaki Y, Masuda M (2010). Transplantation of allogeneic hematopoietic stem cells for adult T-cell leukemia: a nationwide retrospective study. Blood.

[8] Kanda J, Hishizawa M, Utsunomiya A, Taniguchi S, Eto T, Moriuchi Y, Tanosaki R, Kawano F, Miyazaki Y, Masuda M (2012). Impact of graft-versus-host disease on outcomes after allogeneic hematopoietic cell transplantation for adult T-cell leukemia: a retrospective cohort study. Blood.

[9] Ishida T, Joh T, Uike N, Yamamoto K, Utsunomiya A, Yoshida S, Saburi Y, Miyamoto T, Takemoto S, Suzushima H (2012). Defucosylated anti-CCR4 monoclonal antibody (KW-0761) for relapsed adult T-cell leukemia-lymphoma: a multicenter phase II study. J Clin Oncol.

[10] Ishida T, Fujiwara H, Nosaka K, Taira N, Abe Y, Imaizumi Y, Moriuchi Y, Jo T, Ishizawa K, Tobinai K (2016). Multicenter phase II study of Lenalidomide in relapsed or recurrent adult T-cell leukemia/lymphoma: ATLL-002. J Clin Oncol.

[11] Nagano S, Oshika H, Fujiwara H, Komiya S, Kosai K (2005). An efficient construction of conditionally replicating adenoviruses that target tumor cells with multiple factors. Gene Ther.

[12] Horikawa Y, Wang Y, Nagano S, Kamizono J, Ikeda M, Komiya S, Kosai KI (2011). Assessment of an altered E1B promoter on the specificity and potency of triple-regulated conditionally replicating adenoviruses: implications for the generation of ideal m-CRAs. Cancer Gene Ther.

[13] Rodriguez R, Schuur ER, Lim HY, Henderson GA, Simons JW, Henderson DR (1997). Prostate attenuated replication competent adenovirus (ARCA) CN706: a selective cytotoxic for prostate-specific antigen-positive prostate cancer cells. Cancer Res.

[14] Nettelbeck DM, Rivera AA, Balague C, Alemany R, Curiel DT (2002). Novel oncolytic adenoviruses targeted to melanoma: specific viral replication and cytolysis by expression of E1A mutants from the tyrosinase enhancer/promoter. Cancer Res.

[15] Wirth T, Zender L, Schulte B, Mundt B, Plentz R, Rudolph KL, Manns M, Kubicka S, Kuhnel F (2003). A telomerase-dependent conditionally replicating adenovirus for selective treatment of cancer. Cancer Res.

[16] Fukuda S, Pelus LM (2006). Survivin, a cancer target with an emerging role in normal adult tissues. Mol Cancer Ther.

[17] Kamizono J, Nagano S, Murofushi Y, Komiya S, Fujiwara H, Matsuishi T, Kosai K (2005). Survivin-responsive conditionally replicating adenovirus exhibits cancer-specific and efficient viral replication. Cancer Res.

[18] Tanoue K, Wang Y, Ikeda M, Mitsui K, Irie R, Setoguchi T, Komiya S, Natsugoe S, Kosai K (2014). Survivin-responsive conditionally replicating adenovirus kills rhabdomyosarcoma stem cells more efficiently than their progeny. J Transl Med.

[19] Mitsui K, Ide K, Takayama A, Wada T, Irie R, Kosai K (2015). Conditionally replicating adenovirus prevents pluripotent stem cell-derived teratoma by specifically eliminating undifferentiated cells. Mol Ther Methods Clin Dev.

[20] Che XF, Zheng CL, Owatari S, Mutoh M, Gotanda T, Jeung HC, Furukawa T, Ikeda R, Yamamoto M, Haraguchi M (2006). Overexpression of survivin in primary ATL cells and sodium arsenite induces apoptosis by down-regulating survivin expression in ATL cell lines. Blood.

[21] Arima N, Molitor JA, Smith MR, Kim JH, Daitoku Y, Greene WC (1991). Human T-cell leukemia virus type I tax induces expression of the Rel-related family of kappa B enhancer-binding proteins: evidence for a pretranslational component of regulation. J Virol.

[22] Bergelson JM, Cunningham JA, Droguett G, Kurt-Jones EA, Krithivas A, Hong JS, Horwitz MS, Crowell RL, Finberg RW (1997). Isolation of a common receptor for Coxsackie B viruses and adenoviruses 2 and 5. Science (New York, NY).

[23] Teoh G, Chen L, Urashima M, Tai YT, Celi LA, Chen D, Chauhan D, Ogata A, Finberg RW, Webb IJ (1998). Adenovirus vector-based purging of multiple myeloma cells. Blood.

[24] Turturro F, Seth P, Link CJ (2000). In vitro adenoviral vector p53-mediated transduction and killing correlates with expression of coxsackie-adenovirus receptor and alpha (nu)beta5 integrin in SUDHL-1 cells derived from anaplastic large-cell lymphoma. Clin Cancer Res.

[25] Fukuda S, Pelus LM (2001). Regulation of the inhibitor-of-apoptosis family member survivin in normal cord blood and bone marrow CD34(+) cells by hematopoietic growth factors: implication of survivin expression in normal hematopoiesis. Blood.

[26] Shayakhmetov DM, Papayannopoulou T, Stamatoyannopoulos G, Lieber A (2000). Efficient gene transfer into human CD34(+) cells by a retargeted adenovirus vector. J Virol.

[27] Robert C, Long GV, Brady B, Dutriaux C, Maio M, Mortier L, Hassel JC, Rutkowski P, McNeil C, Kalinka-Warzocha E (2015). Nivolumab in previously untreated melanoma without BRAF mutation. N Engl J Med.

[28] Reck M, Rodriguez-Abreu D, Robinson AG, Hui R, Csoszi T, Fulop A, Gottfried M, Peled N, Tafreshi A, Cuffe S (2016). Pembrolizumab versus chemotherapy for PD-L1-positive non-small-cell lung Cancer. N Engl J Med.

[29] Motzer RJ, Escudier B, McDermott DF, George S, Hammers HJ, Srinivas S, Tykodi SS, Sosman JA, Procopio G, Plimack ER (2015). Nivolumab versus Everolimus in advanced renal-cell carcinoma. N Engl J Med.

[30] Harrington KJ, Ferris RL, Blumenschein G, Colevas AD, Fayette J, Licitra L, Kasper S, Even C, Vokes EE, Worden F (2017). Nivolumab versus standard, single-agent therapy of investigator's choice in recurrent or metastatic squamous cell carcinoma of the head and neck (CheckMate 141): health-related quality-of-life results from a randomised, phase 3 trial. Lancet Oncol.

[31] Todo T, Rabkin SD, Sundaresan P, Wu A, Meehan KR, Herscowitz HB, Martuza RL (1999). Systemic antitumor immunity in experimental brain tumor therapy using a multimutated, replication-competent herpes simplex virus. Hum Gene Ther.

[32] Andtbacka RH, Kaufman HL, Collichio F, Amatruda T, Senzer N, Chesney J, Delman KA, Spitler LE, Puzanov I, Agarwala SS (2015). Talimogene Laherparepvec improves durable response rate in patients with advanced melanoma. J Clin Oncol.

[33] Rooney MS, Shukla SA, Wu CJ, Getz G, Hacohen N (2015). Molecular and genetic properties of tumors associated with local immune cytolytic activity. Cell.

[34] Kataoka K, Shiraishi Y, Takeda Y, Sakata S, Matsumoto M, Nagano S, Maeda T, Nagata Y, Kitanaka A, Mizuno S (2016). Aberrant PD-L1 expression through 3′-UTR disruption in multiple cancers. Nature.

